# Levels of Produced Antibodies after Vaccination with mRNA Vaccine; Effect of Previous Infection with SARS-CoV-2

**DOI:** 10.3390/jcm10132842

**Published:** 2021-06-27

**Authors:** Theocharis G. Konstantinidis, Stavroula Zisaki, Ioannis Mitroulis, Eleni Konstantinidou, Eftychia G. Kontekaki, Gioulia Romanidou, Alexandros Karvelas, Ioanna Nanousi, Leonidas Lazidis, Dimitrios Cassimos, Christina Tsigalou, Georges Martinis, Maria Panopoulou

**Affiliations:** 1 Blood Transfusion Center, University General Hospital of Alexandroupolis Dragana Campus, 68100 Alexandroupolis, Greece; stavzi@yahoo.gr (S.Z.); kontekaki_e@hotmail.com (E.G.K.); ionanousi@yahoo.gr (I.N.); tsetsenos258@yahoo.gr (L.L.); geomimar@gmail.com (G.M.); 2 Laboratory of Microbiology, Democritus University of Thrace, University General Hospital of Alexandroupolis Dragana Campus, 68100 Alexandroupolis, Greece; alexnet.gr@gmail.com (A.K.); ctsigalo@med.duth.gr (C.T.); mpanopou@med.duth.gr (M.P.); 3 First Department of Internal Medicine, Democritus University of Thrace, 68100 Alexandroupolis, Greece; imitroul@med.duth.gr; 4 Blood Transfusion Center, General Hospital of Drama, 66100 Drama, Greece; helenkonst28@gmail.com; 5 Nephrology Department, General Hospital “Sismanogleio”, 69100 Komotini, Greece; dr_giouliarom@yahoo.gr; 6 Pediatric Department, Democritus University of Thrace, 68100 Alexandroupolis, Greece; dkasimos@med.duth.gr

**Keywords:** vaccination, COVID-19, anti-SARS-CoV-2 antibodies, SARS-CoV-2

## Abstract

The aim of this study was to estimate the immunogenic effect of mRNA vaccine against SARS-CoV-2. This study included 510 participants who received mRNA vaccine. The measurement of anti-COVID-19 antibodies was performed using the Abbott SARS-CoV-2 IgG quantitative assay (Abbott). Overall, mean titer of anti-Spike antibodies was 19,319.2 ± 1787.5 AU/mL. Vaccination induced a robust immunogenic response in those previously infected with SARS-CoV-2 compared with non-infected subjects. Additionally, individuals that were asymptomatic after vaccination produced lower levels of antibodies compared to feverish individuals. In conclusion, remarkably high levels of anti-Spike COVID-19 antibodies were observed after vaccination.

## 1. Introduction

The novel Coronavirus Disease (COVID-19) that is a result of the Severe Acute Respiratory Syndrome Coronavirus 2 (SARS CoV-2) is an emerging global health problem that was firstly reported in Wuhan, China, in December 2019 [1]. Due to person-to-person transmission, the infection was rapidly spread worldwide.

Vaccination is a safe and effective method for protection against infectious diseases. Vaccines train the immune response to recognize and neutralize an infectious agent. On account of the long-standing vaccination programs during childhood and adulthood, large epidemics of infectious diseases have reduced worldwide [2].

For the SARS-CoV-2 pandemic control, it is fundamental to develop vaccines and to rapidly organize their implementation. There are multitudes of anti-SARS-CoV-2 vaccine candidates, based on several different mechanisms of action that are currently in development or finished phase III trials [3,4]. Since December 2020, several vaccines have been authorized worldwide. In Greece, vaccination was started from Healthcare professionals and on 13 March 2021, 1,283,472 doses of vaccines were used [5].

The aim of this study was to estimate the seroprevalence of SARS-CoV-2 antibodies among persons who took two doses of mRNA vaccine in the Thrace region and to explore risk factors for any adverse events.

## 2. Materials and Methods

### 2.1. The Study Design

This study was performed at the University General Hospital of Alexandroupolis and Democritus University of Thrace in Alexandroupolis, Greece. The study protocol was approved by the local committee of ethics and deontology in accordance with the Declaration of Helsinki (Number 1070/11-01-2021).

### 2.2. Study Population

This study included 510 persons, which includes both uninfected (*n* = 487) and previously infected persons (*n* = 23) with confirmed COVID-19 by RT-PCR occurring 2 to 3.5 months prior to vaccination. All persons were vaccinated with mRNA (BNT162b2 Pfizer/BioNTech) vaccine. Serum samples were collected before vaccination and 1 month after the second dose. The period of sampling was from 10 February 2021 to 16 March 2021.

### 2.3. IgG Testing

The measurement of anti-COVID-19 antibodies was performed using the Abbott Architect i1000SR instrument (Abbott Diagnostics, Abbott Park Road, IL, USA) and the Abbott SARS-CoV-2 IgG quantitative kit by following the manufacturer’s instructions. The assay is a chemiluminescent microparticle immunoassay for the qualitative detection of anti-SARS-CoV-2 Abs type IgG against the CoV-2 Spike protein (Sp) in human serum. Quantitative results > 50 AU/mL are reported as positive in accordance with the Abbott-determined positivity cutoff of 50 AU/mL.

### 2.4. Statistical Analysis

Continuous variables were presented as mean ± standard deviation (SD) for normally distributed data. The counting data were expressed by rate (%). The Mann–Whitney U test was used for independent samples and the Wilcoxon test was used for paired sample analysis. The *p* value < 0.05 indicated a statistically significant difference.

## 3. Results

### 3.1. Safety Assesment

Overall, 510 persons were enrolled in the study. The demographic data is presented in Table 1. Summary data (numbers and percentages) for participants with any adverse events reflected that the most common were local adverse events. The injection-site event was pain after injection and this was noted in 114 participants (22.5%) after the first dose and/or the second dose and the pain resolved over 1 to 5 days. The most common system adverse events were fever—95 (18.6%); headache—78 (15.3%); myalgias—68 (13.3%); arthralgia—12 (2%); fatigue—57 (11.2%); and lymphadenopathy—22 (4.3%) (Table 1). The severe adverse events resulting in the discontinuation of the second dose injections were recorded in 3/510 (0.59%) participants who presented with severe allergic reactions. Only one woman developed delayed hypersensitivity reaction and the reaction initially started as macular on the fourth day after vaccination, but subsequently developed maculopapular lesions with symmetrical distribution on the extremities.

### 3.2. The Immunogenic Effect

**Figure 1:**
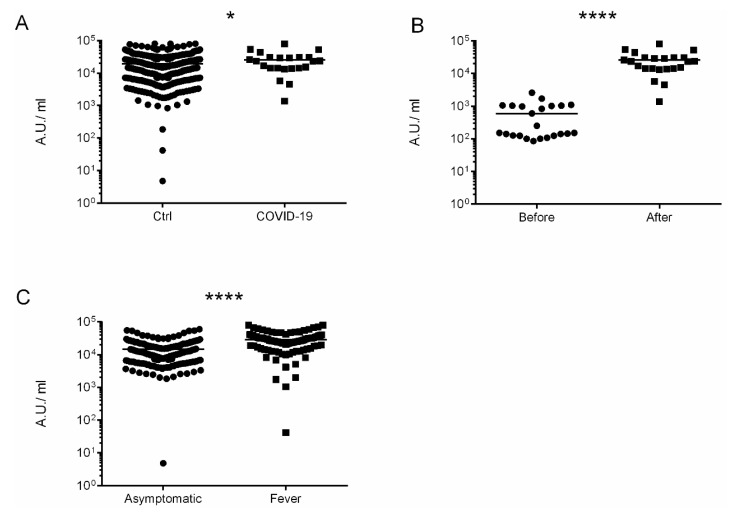
The immunogenic effect of mRNA vaccine. (**A**) Levels of antibodies after vaccination. Comparison of controls vs. COVID-19 patients. (**B**) Levels of antibodies in COVID-19 patients before and after vaccination. (**C**) Levels of antibodies after vaccination in asymptomatic persons vs. systemic adverse events (fever) persons. The date is presented as log10. * *p* = 0.049 and **** *p* < 0.0001.

Overall, the immunization with administration of two doses were completed in 507 participants (99.4%). Anti-Spike SARS-CoV-2-IgG antibodies were detected in 508 out of 510 (99.6%), with the mean value 19,319.2 ± 1787.5 AU/mL. Participants who suffered from COVID-19 had higher levels of anti-Spike antibodies in comparison to non-infected 25,599.5 ± 10,646.8 vs. 19,221.3 ± 1803.66, *p* = 0.049, respectively (Figure 1A). Moreover, COVID-19 patients had developed higher levels of anti-Spike antibodies after vaccination 593.7 ± 379.2 vs. 25,599.5 ± 10,646.8 AU/mL, *p* < 0.00001 (Figure 1B). Patients with fever developed higher titer of anti-Spike Abs in comparison to asymptomatic participants (28,899.6 ± 4831.01 vs. 14,685.9 ± 214.1 *p* < 0.00001) Figure 1C. Moreover, patients with autoimmune disorders had lower titer of anti-Spike Abs than the general population in a statistically significant manner 6311.18 ± 557.1 vs. 19,319.2 ± 1787.5 AU/mL.

## 4. Discussion

This study provides data on the magnitude of IgG titers after mRNA vaccination in an adult population in the Thrace region, Greece. Overall, in this study, only three severe adverse events occurring after the receipt of the first vaccine that led to postponing the second dose has been reported. The adverse events after the receipt of Pfizer/BioNTech COVID-19 vaccine in the United States were reported in 4393 (0.2%) cases. Among these, cases of severe allergic reaction, including anaphylaxis, were recorded [6]. In this study the association of systemic symptoms, such as fever with higher IgG responses to Spike, was observed. These results are in agreement with previously reported studies. The cases of the acute onset of a single lymphadenopathy (supraclavicular or Axillary) after intramuscular administration of an mRNA-based COVID-19 vaccine recorded in 20 participants in this study. These results are in line with what is previously reported by O R Mitchell et al. and Fernández-Prada et al. [7,8]. The mean value of anti-SARS-CoV-2 Spike protein in patients with autoimmune disorders in this study was lower than in the general population (6311.18 ± 557.1 vs 19,319.2 ± 1787.5 AU/mL). The data on specific COVID-19 vaccine responses in patients under immunosuppressive therapy have been poorly documented until now. Immunosuppressive therapy in patients with autoimmune disorders or transplantation may impair vaccine responses. These data were previously shown upon vaccination of immunosuppressive patients and predominantly focuses on influenza and pneumococcal vaccines [9]. A limitation of the study is the relatively small group of vaccinated patients after SARS CoV infection.

## Figures and Tables

**Table 1:** Demographical, clinical, and adverse events data of study population.

	Non-Infected *n* = 487	COVID-19 *n* = 23	Overall *n* = 510
Age	48.4 ± 2.5	47.1 ± 2.3	47.5 ± 2.5
Male	147 (30.2%)	6 (26.1%)	153 (30%)
Female	340 (69.8%)	17 (73.9%)	357 (70%)
Occupational Risk			
Healthcare Workers	457 (93.8%)	23 (100%)	480 (94%)
Underlying disease			
CVD	18 (3.7%)	1 (4.3%)	19 (3.7
Hypertension	22 (4.5%)	2 (8.3%)	24 (4.7%)
Diabetes mellitus	17 (3.5%)	2 (8.3%)	19 (3.7%)
Cancer	6 (1.2%)		6 (1.2%)
Autoimmune diseases	15 (3.1%)		15 (2.9%)
Adverse events			
No Adverse Events	184 (38.1%)	5 (21%)	189 (37%)
Solicited Local			
Pain	106 (21.9%)	8 (34.8%)	114 (22.5%)
Swelling			
Lymphadenopathy	20 (4.1%)	2 (8.7%)	22 (4.3%)
Supraclavicular	3 (0.6%)	-	3 (0.59%)
Axillary	17 (3.5%)	2 (8.7%)	19 (3.75%)
Systemic Adverse Events			
Fever	90 (18.5%)	5 (21.7%)	95 (18.2%)
Shiver	79 (16.2%)	5 (21.7%)	84 (16.5%)
Headache	74 (15.9%)	4 (17.4%)	78 (15.3%)
Fatigue	54 (11.1%)	3 (13%)	57 (11.2%)
Nausea/vomiting	15 (3.1%)		15 (2.9%)
Myalgia	64 (13.1%)	4 (17.4%)	68 (13.3%)
Arthralgia	8 (1.6%)	4 (17.4%)	12 (2.4%)
Hypersensitivity			
Type IV hypersensitivity reaction	1 (0.2%)		1 (0.19%)
Edema	3 (0.6%)		3 (0.59%)

## Data Availability

The dataset generated during the study contains sensitive personal information. Some of this data is available from the corresponding author on reasonable request.
